# 成人系统性肥大细胞增多症诊断与治疗中国指南（2022年版）

**DOI:** 10.3760/cma.j.issn.0253-2727.2022.12.001

**Published:** 2022-12

**Authors:** 中华医学会血液学分会实验诊断学组 中国肥大细胞增多症协作网络

系统性肥大细胞增多症（systemic mastocytosis，SM）是一种具有独特病理、临床特征和分子表达的髓系肿瘤类型。长期以来，其诊断和治疗尚未得到国内血液病同行的足够重视。为了进一步推广我国SM的规范化临床诊治流程，由中华医学会血液学分会实验诊断学组和中国肥大细胞增多症协作网络工作组牵头，在广泛征求国内专家意见的基础上，制订了本指南。

一、定义

1. 肥大细胞增多症是异常肥大细胞在一个或多个组织器官中浸润导致的一种罕见的异质性肿瘤。根据第五版世界卫生组织（WHO）分类标准，肥大细胞增多症可分为三个疾病亚型——皮肤型肥大细胞增多症（CM）、SM和肥大细胞肉瘤（MCS）[1]。

2. SM是侵袭性肥大细胞在真皮组织以外的一个或多个组织、器官或系统的克隆性增殖，并释放大量的血管活性介质，引起多器官功能障碍，常见的受累部位包括骨髓、皮肤、脾脏、淋巴结、肝脏和（或）单核-巨噬细胞系统，约占肥大细胞增多症的10％[2]。

二、诊断程序

1. 病史采集：详细的病史采集和体格检查对明确病因、评价肿瘤负荷及脏器受累情况极其重要。明确既往有无肥大细胞活化的症状，包括是否有皮肤肥大细胞增多的症状，是否有过敏性疾病、淋巴结肿大、关节肌肉疼痛、心肺和胃肠道症状，是否有乏力、发热、出汗、瘙痒、体重下降和酒精相关疼痛等介质症状。

2. SM临床表现：包括全身症状、肥大细胞活化症状（介质导致的症状）、相关疾病症状以及体征：

（1）全身症状：发热、疲劳、体重减轻、盗汗等。

（2）肥大细胞活化症状：①过敏反应；②乏力；③眩晕、晕厥/昏迷；④皮肤：面部、颈部和胸部皮肤潮红，瘙痒伴或不伴皮疹，荨麻疹伴或不伴血管性水肿；⑤胃肠：胃部不适、腹泻、恶心、呕吐、腹痛、腹胀、胃食管反流病；⑥神经精神：头痛、脑雾、认知障碍、焦虑、抑郁、记忆力减退、注意力不集中；⑦心血管：心率快、胸痛、低血压、高血压、血压不稳定；⑧呼吸：喘息和气短；⑨骨骼肌肉：骨骼肌肉疼痛、骨质硬化、骨质减少、骨质疏松；⑩鼻喉：鼻痒、鼻塞、喉咙发痒和肿胀。

（3）相关疾病症状：部分患者存在血液系统疾病相关症状。

（4）体征：肝肿大、脾肿大和淋巴结肿大等。

（5）B-发现和C-发现[3]（表1）：B-发现提示：肥大细胞负荷高，肿瘤进展累及多系造血，但没有器官功能障碍；C-发现提示：肥大细胞浸润造成的器官功能损害（尽量行活检证实）。

**表1:** 系统性肥大细胞增多症（SM）的B-发现（疾病负荷）和C-发现（器官受累）

B-发现（提示肥大细胞负荷高，但无器官损害）	C-发现^a^（提示SM诱导的器官损害）
高肥大细胞负荷：	
骨髓病理免疫组织化学显示骨髓中肥大细胞浸润≥30%和（或）血清类胰蛋白酶≥200 ng/ml和（或）骨髓或外周血白细胞中KIT D816V VAF≥10%	-
骨髓增殖和（或）骨髓发育异常^b^：	血细胞减少（至少发现一项）：
骨髓有核细胞增多活跃伴脂肪细胞丢失和显著的髓系造血±核左移且嗜酸性粒细胞增多±白细胞增多和嗜酸性粒细胞增多和（或）散在的发育异常征象（中性粒细胞、红细胞以及巨核细胞的发育异常<10%）	中性粒细胞绝对值<1.0×10^9^/L；
	HGB<100 g/L；
	PLT<100×10^9^/L
器官肿大：	器官肿大伴功能损害：
可触及的肝肿大，无腹水和其他肝功能损害征象和（或）可触及的脾肿大，无脾功能亢进和体重减轻和（或）可触及的淋巴结病变或超声、CT发现肿大淋巴结	肝：腹水且肝酶升高±肝肿大或肝硬化±门静脉高压；
	脾：可触及的脾肿大伴脾功能亢进±体重减轻±低白蛋白血症；
	胃肠道：吸收不良伴低白蛋白血症±体重减轻；
	骨骼：大面积（≥2 cm）溶骨性病变伴病理性骨折±骨痛

注 ^a^由合并的相关血液肿瘤（AHN）或其他病因（如感染或治疗相关）引起的器官损害不能被认为是C-发现；局部SM浸润应尽量通过活检证实；^b^骨髓增殖和骨髓发育异常在随访过程中既不消失也不进展，且不能达到骨髓增殖性肿瘤（MPN）、骨髓增生异常综合征（MDS）或MDS/MPN的诊断标准；否则，SM亚型应该诊断为SM合并相关血液肿瘤（SM-AHN）。AHN部分诊断髓系肿瘤时，B-发现和冒烟型SM中髓系造血相关的定义不应考虑在内

3. 实验室检查：

（1）血液学检查：可见贫血、白细胞增多、单核细胞增多、中性粒细胞减少和血小板减少等，嗜酸性粒细胞增多亦较常见。肥大细胞白血病（MCL）患者外周血可检出肥大细胞。

（2）血清类胰蛋白酶：类胰蛋白酶从分泌颗粒中大量释放是肥大细胞脱颗粒的特征。基线血清总类胰蛋白酶持续高于20 ng/ml是诊断SM的次要标准。SM合并相关血液肿瘤（SM-AHN）和侵袭性SM（ASM）患者的血清类胰蛋白酶水平多显著升高（>200 ng/ml）。当同时诊断髓系肿瘤时，基线血清总类胰蛋白酶水平不适用于SM标准。

（3）骨髓形态学检查：正常肥大细胞呈圆形、卵圆形，胞核居中，胞质颗粒充盈；不典型肥大细胞可呈梭形、偏位核，胞质颗粒减少；幼稚肥大细胞呈锯齿状、分叶状核，原始肥大细胞胞质中可见异染颗粒。应明确异常肥大细胞占全部肥大细胞的百分比，穿刺标本应关注相关血液肿瘤（AHN）的特征。

（4）骨髓组织病理学[4]：应对肥大细胞聚集程度进行判定，关注肥大细胞浸润方式（多灶性致密浸润或弥散性间质浸润）。免疫组织化学标记应包括类胰蛋白酶、CD117、CD25、CD2和CD30。类胰蛋白酶阳性是肥大细胞的特异性抗原标记，但类胰蛋白酶和CD117不能区分正常和肿瘤性肥大细胞。CD30在大多数SM患者的肿瘤性肥大细胞中表达，在其他髓系肿瘤中未发现。

（5）流式细胞术：CD117、CD25、CD2和CD30是流式细胞术检测SM的标准标志物。CD2和CD25的异常表达有助于鉴别SM中的肥大细胞与正常肥大细胞和反应性肥大细胞（表2）。

**表2:** 系统性肥大细胞增多症（SM）的鉴别诊断[7]

疾病	细胞浸润模式	免疫表型	KIT D816V
SM	致密浸润（符合SM）	Typtase（+），CD117（+），CD25/CD2/CD30（+）	+
急性嗜碱性粒细胞白血病	间质性浸润	Typtase（弱+），CD117（−/+），CD13/CD33（+），CD123（+），CD203c（+），BB1（+），2D7（+）	−
反应性肥大细胞增多	间质性浸润	Typtase（+），CD117（+），CD25/CD2/CD30（−）	−
髓系肿瘤伴嗜酸性粒细胞增多且PDGFRA/PDGFRB重排	间质性浸润或聚集成簇	Typtase（+），CD117（+），CD25（+）	−
粒-肥大细胞白血病	肥大细胞疏松分散性间质性浸润，白血病细胞间质性浸润	肥大细胞：Typtase（+），CD117（+），CD25/CD2（−）；白血病细胞：CD34（+），CD25（−），CD117（+）	−
类胰蛋白酶阳性AML	间质性浸润	Typtase（+），CD117（+），CD25（−），CD34（+），CD13/33（+）	+/−

（6）细胞遗传学检查：应采用G显带或R显带技术进行核型分析。目前SM的细胞遗传学研究数据较少。来自美国梅奥诊所348例SM的数据显示，15％的SM患者可检出核型异常［惰性系统性肥大细胞增多症（ISM）核型异常检出率为6％，SM-AHN核型异常检出率为26％，ASM核型异常检出率为8％］[5]。

（7）分子学检查：KIT突变阳性为SM次要诊断标准之一。90％以上的SM患者可检出KIT基因突变，以KIT D816V突变最为常见，其他常见异常类型包括KIT D816H/Y、N822K等。此外，进展型SM还可检出TET2、SRSF2、CBL、ASXL1、RUNX1、EZH2、RAS等基因突变[6]。

三、诊断

SM可分为骨髓肥大细胞增多症（BMM）、ISM、冒烟型系统性肥大细胞增多症（SSM）、ASM、SM-AHN和MCL六个亚型（图1）。SM需满足主要标准和1项次要标准或同时满足≥3项次要标准（表3）。

**图1:**
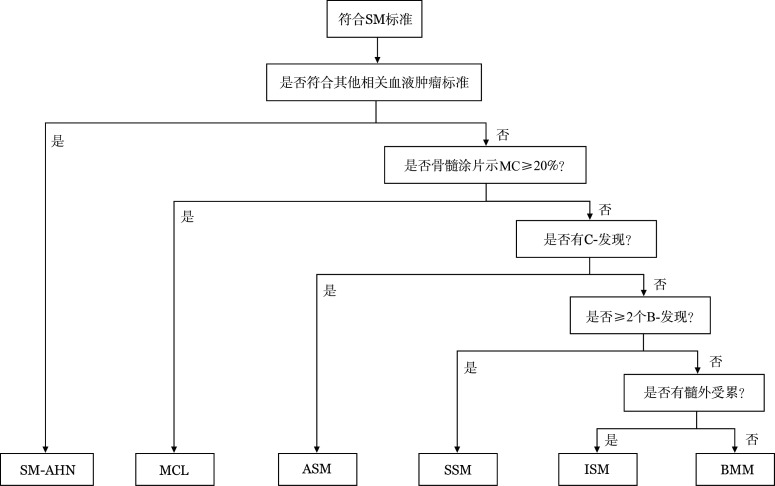
系统性肥大细胞增多症（SM）各亚型诊断流程 注 SM：系统性肥大细胞增多症；MC：肥大细胞；SM-AHN：系统性肥大细胞增多症合并相关血液肿瘤；MCL：肥大细胞白血病；ASM：侵袭性系统性肥大细胞增多症；SSM：冒烟型系统性肥大细胞增多症；ISM：惰性系统性肥大细胞增多症；BMM：骨髓肥大细胞增多症；SM-AHN需满足一种相关血液肿瘤，MCL内部还可以分为不同亚型，ASM常无皮损，SSM具有高肥大细胞负荷特点，ISM皮损常见，但肥大细胞负荷低，BMM常无B-发现，基线血清类胰蛋白酶水平<125 ng/ml

**表3:** 系统性肥大细胞增多症的诊断标准

标准	描述
主要标准	骨髓组织切片或其他非皮肤器官切片中肥大细胞呈多灶性致密浸润（一簇中聚集的肥大细胞数≥15个）
次要标准	①骨髓涂片中肥大细胞>25％为不典型形态（Ⅰ型或Ⅱ型）或骨髓组织/非皮肤器官切片中浸润的肥大细胞中>25％为梭形^a^；
	②骨髓或其他皮外器官中检测到KIT基因密码子816或其他明确具有病理意义位点的突变（密码子417，501–509，522，557–560，642，654，799，816，820，822等）；
	③骨髓、血液或其他非皮肤器官中肥大细胞表达CD2和（或）CD25和（或）CD30^b^；
	④基线血清总类胰蛋白酶持续>20 ng/ml[合并髓系肿瘤时暂不适用，合并遗传性α-胰蛋白酶血症（HαT）时，标准应校正^c^]

注 ^a^在组织切片中，形态异常应当计数致密浸润和弥漫浸润的肥大细胞。然而，当肥大细胞内衬血管细胞、脂肪细胞、神经细胞或内膜内皮细胞层时，梭形形态不作为SM的标准。在骨髓涂片中，当肥大细胞位于或邻近骨髓小粒时，肥大细胞的非典型形态不可作为SM的标准。^b^当通过流式细胞术或免疫组化两种技术来证实肥大细胞表达时，所有3个标记物均需符合这一次要的SM标准。^c^虽然最佳的校正方法仍需要确定，但有一种方法是：校正的血清类胰蛋白酶水平=基础血清类胰蛋白酶水平/（1+类胰蛋白酶基因的额外拷贝数）。如当血清类胰蛋白酶水平为300，并且在HαT患者中发现2个额外的类胰蛋白酶基因拷贝时，HαT校正的血清类胰蛋白酶水平为100（300/3=100），因此不符合B-发现

1. BMM：

（1）符合SM的诊断标准；

（2）无B-发现；

（3）无C-发现；

（4）无AHN、MCL的证据；

（5）髓外器官中无肥大细胞致密浸润；

（6）基线血清类胰蛋白酶水平<125 ng/ml；

（7）无皮肤损害。

2. ISM（ISM包括典型ISM及不伴有皮肤浸润的ISM）：

（1）典型ISM：①符合SM的诊断标准；②有≤ 1项B-发现；③无C-发现；④无AHN、MCL证据；⑤有典型皮肤损害。

对于伴有皮肤损害，而未能进行骨髓检查的成人患者，皮肤肥大细胞增多（MIS）可作为临时诊断，这类患者表现为皮肤损害、Daier's征阳性，皮肤或外周血KIT突变可为阳性或阴性，不伴其他SM的器官损害。对于有肥大细胞脱颗粒症状并伴有克隆性/肿瘤性肥大细胞［即KIT突变和（或）CD25表达］的患者，如果不符合SM标准（仅满足1到2个次要标准，且无皮肤受累），考虑为“诊断前ISM（pre-diagnostic ISM）”或“单克隆肥大细胞活化综合征（MMAS）”，其血清类胰蛋白酶水平多正常或轻度升高（图2）。

**图2:**
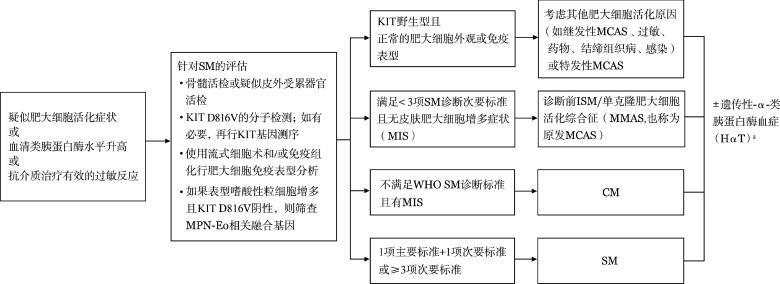
临床疑似肥大细胞增多症患者的诊断流程[8] 注 SM：系统性肥大细胞增多症；MPN-Eo：骨髓增殖性肿瘤伴嗜酸性细胞增多；MCAS：肥大细胞活化综合征；ISM：惰性系统性肥大细胞增多症；CM：皮肤肥大细胞增多症；^a^HαT可与MCAS、SM、CM等肥大细胞疾病合并存在，也可单独存在，常见于SM，尤其是ISM和SSM，与严重介质症状/过敏反应风险增加相关

（2）不伴有皮肤浸润的ISM：①符合SM的诊断标准；②有≤1项B-发现和（或）基线血清类胰蛋白酶水平≥125 ng/ml和（或）髓外器官中肥大细胞致密浸润；③无C-发现；④无AHN、MCL证据；⑤无皮肤损害。

3. SSM：

（1）符合SM的诊断标准；

（2）有≥2项B-发现；

（3）无C-发现；

（4）无AHN、MCL证据。

4. ASM：

（1）符合SM的诊断标准；

（2）有≥1项C-发现；

（3）不符合MCL的诊断标准；

（4）通常无皮肤损害。

根据既往有无肥大细胞肿瘤病史分为原发性ASM与继发性ASM。

5. SM-AHN：

（1）符合SM的诊断标准；

（2）符合相关血液肿瘤的诊断标准（除外BMM合并的Ⅰ期外周淋巴结或单个器官淋巴瘤）。

根据SM不同亚型，可分为BMM-AHN、ISM-AHN、SSM-AHN、ASM-AHN和MCL-AHN；根据AHN不同类型，可以分为SM-AML、SM-CMML、SM-ALL、SM-MM等。

在SM-AHN中，肥大细胞浸润可能被AHN细胞浸润掩盖，因此SM-AHN诊断常在细胞消减后才可确立。因此对于伴有KIT D816V突变的髓系肿瘤患者建议在细胞消减后再次进行SM的诊断。

6. MCL：

（1）符合SM的诊断标准；

（2）骨髓活检示不典型、幼稚肥大细胞弥漫浸润（常致密浸润）；

（3）骨髓涂片示肥大细胞≥20％；

（4）通常无皮肤损害。

MCL亚型包括：①急性MCL（≥1个C-发现）与慢性MCL（无C-发现）；②原发性MCL与继发性MCL（继发于其他SM亚型甚至肥大细胞肉瘤）；③白血病性MCL（外周血白细胞中肥大细胞≥10％）与非白血病性MCL（外周血白细胞中肥大细胞<10％，更为常见）；④MCL-AHN与MCL不伴AHN。

此外，分化良好的SM（WDSM）是一种可发生在任何SM亚型中的形态学模式，其特征是圆形和颗粒丰富的肥大细胞明显浸润骨髓。在多数WDSM患者中，未检测到KIT密码子816突变，肿瘤性肥大细胞的CD25和CD2通常为阴性，而CD30为阳性。

四、预后和危险分层：

1. 不良预后因素：

（1）临床/实验室因素：

①进展型系统性肥大细胞增多症（AdvSM）（包括ASM、SM-AHN、MCL）；

②高龄、体重减轻、贫血、血小板减少、低白蛋白血症和骨髓原始细胞增多（>5％）；

③嗜酸性粒细胞增多^a^；

④脾大；

⑤碱性磷酸酶水平升高；

⑥血浆IL2Rα/CD25水平升高[9]。

（2）细胞遗传学/分子学因素：

①不良预后核型（单体7或复杂核型）[10]；

②KIT-D816V突变多系受累；

③非KIT-D816V突变基因的数目；

④SRSF2/ASXL1/RUNX1（S/A/R）或EZH2/ASXL1/CBL突变^b^。

注：^a^KIT D816V突变阴性或有嗜酸性粒细胞增多表现且携带FIP1L1-PDGFRA融合基因的患者预后良好；^b^ASXL1和CBL突变对晚期SM患者生存不良有独立预测作用。

2. 危险分层：

（1）肥大细胞增多症国际预后评分系统（International Prognostic Scoring System For Mastocytosis，IPSM）适用于所有SM患者的危险度分层（表4）。

**表4:** 肥大细胞增多症国际预后评分系统（IPSM）

类型	预后变量	积分
非AdvSM^a^	年龄≥60岁	1
	血清碱性磷酸酶≥100 U/L	1
AdvSM^b^	年龄≥60岁	1
	类胰蛋白酶≥125 ng/ml	1
	WBC≥16×10^9^/L	1
	HGB≤110 g/L	1
	PLT≤100×10^9^/L	1
	皮肤受累	−1

注 AdvSM：进展型系统性肥大细胞增多症；^a^非AdvSM的危险分层：低危：0分；中危-1：1分；中危-2：2分。^b^AdvSM的危险分层：AdvSM-1：−1～0分；AdvSM-2：1分；AdvSM-3：2～3分；AdvSM-4：4～5分

（2）梅奥联盟预后系统（Mayo Alliance Prognostic System，MAPS）适用于所有肥大细胞增多症患者的危险度分层（表5）。

**表5:** 梅奥联盟预后系统（MAPS）

预后变量	积分
年龄>60岁	1
AdvSM（非ISM/SSM）	2
PLT<150×10^9^/L	1
血清碱性磷酸酶大于正常范围	1
不良预后突变（ASXL1，RUNX1，NRAS）	1

注 AdvSM：进展型系统性肥大细胞增多症；低危：≤2分；中危-1：3分；中危-2：4分；高危：≥5分

（3）AdvSM的突变校正危险度评分（Mutation-Adjusted Risk Score，MARS）适用于AdvSM患者的危险度分层（表6）。

**表6:** AdvSM的突变校正危险度评分（MARS）

预后变量	积分
年龄>60岁	1
HGB<100 g/L	1
PLT<100×10^9^/L	1
存在SRSF2/ASXL1/RUNX1（S/A/R）中的一种突变	1
≥2个S/A/R突变	2

注 AdvSM：进展型系统性肥大细胞增多症；低危：0～1分；中危-1：2分；高危：3～5分

五、治疗

1. 肥大细胞活化症状的抗介质治疗[11]：

（1）避免诱因；

（2）局部应用润肤霜或色甘酸钠护理皮肤；

（3）孤立肥大细胞瘤可考虑手术切除（例如：屈侧、足底、手掌或头皮的病变）；

（4）严重或危及生命的症状可应用抗组胺药、抗白三烯药物、皮质类固醇激素。

2. 慢性肥大细胞介质相关症状的逐步预防性治疗：

如出现皮肤、胃肠道、神经系统、心血管系统、呼吸系统及眼鼻等慢性介质症状，可以采用逐步预防治疗：局部应用色甘酸钠（1％～4％膏/软膏）、酮替芬、阿司匹林、白三烯受体拮抗剂、质子泵抑制剂、H1和H2受体阻断药、奥马珠单抗（Omalizumab）、皮质类固醇激素。

3. 过敏反应的紧急治疗（包括膜翅目毒液过敏反应）：

系统性荨麻疹可应用抗组胺药；系统性荨麻疹急性发作反应且继发其他器官受累（如气道、胃肠、神经、心血管）或急性过敏反应合并危及生命的症状（低血压、喉头水肿、血流动力学障碍、氧饱和度下降、癫痫）时，立即应用肾上腺素（0.3～0.5 mg/剂）肌肉注射（在临床没有改善的情况下每5 min重复3次）；在肌肉注射3剂肾上腺素后可静脉注射肾上腺素。

4. 骨量减少/骨质疏松的治疗。

5. 传统治疗：

（1）羟基脲：作用原理主要在于骨髓抑制作用，对肥大细胞无实质性作用。

（2）干扰素-α：可改善肥大细胞脱颗粒症状，减少骨髓肥大细胞浸润，改善肥大细胞增多症相关腹水/肝脾肿大、血细胞减少、皮疹和骨质疏松。主要反应率为20％～30％；联合皮质类固醇（泼尼松）可改善其疗效和耐受性。

（3）克拉屈滨（2-CdA）：克拉屈滨对肥大细胞肿瘤的治疗作用已被体内外研究所证实。美国梅奥诊所的一项临床研究显示，克拉屈滨治疗SM的平均起效时间为11（3～74）个月，总反应率为55％。

注：克拉屈滨和聚乙二醇干扰素α-2a一般只推荐用于进展型SM患者。但是当患者有严重难治性的介质症状或对抗介质治疗或双膦酸盐无效的骨病，在ISM或SSM患者中也可能有效。

6. 靶向治疗[12]：近年来，靶向KIT的小分子激酶抑制剂的疗效令人鼓舞。

（1）阿伐替尼（Avapritinib，BLU-285）：阿伐替尼可强效、高选择性地抑制KIT exon17活化环突变（包括KIT D816V）和PDGFRA类似突变（包括18号外显子的D842V），获FDA批准用于治疗成人AdvSM患者，推荐剂量为200 mg/d，口服给药。Ⅰ期EXPLORER试验表明，48例可评估的AdvSM患者的总反应率（ORR）为77％，其中85％的肿瘤患者骨髓肥大细胞被清除，92％的患者KIT D816V突变水平降低≥50％。Ⅱ期PATHFINDER试验的中期分析包括32例可评价的AdvSM患者，ORR为75％，同时患者的肥大细胞疾病负荷客观指标也显著降低；3级以上中性粒细胞减少症、贫血和血小板减少症的发生率分别为24％、16％和16％。最常见的3级以上非血液学不良事件为外周/眶周水肿，发生率为3％。

（2）瑞派替尼（Ripretinib，DCC-2618）：Ⅱ型激酶开关口袋调控抑制剂能有效抑制对常规激酶抑制剂耐药的KIT基因17号外显子突变。一项针对瑞派替尼在AdvSM患者中的安全性和耐受性（NCT02571036）的Ⅰ期爬坡试验临床研究正在进行。

（3）米哚妥林（Midostaurin）：可靶向野生型和突变型KIT，两项临床研究的数据显示，晚期SM患者的ORR为60％。米哚妥林的起始量为100 mg，每日2次（不能耐受者减量至50 mg，每日2次），随餐服用。米哚妥林与强CYP3A抑制剂联合使用可能会增加米哚妥林的浓度。

（4）其他酪氨酸激酶抑制剂：甲磺酸伊马替尼、达沙替尼和马赛替尼（Masatinib）对KIT基因常见的D816V突变无效。

7. 异基因造血干细胞移植（allo-HSCT）：

allo-HSCT是目前唯一可以根治SM的治疗方式，对于AdvSM患者应评估allo-HSCT的可行性。一项国际多中心回顾性临床研究显示，AdvSM患者接受allo-HSCT后3年总生存率57％，其中SM-AHN为74％，ASM为43％，MCL为17％[13]。

8. SM各亚型的治疗（图3）：

**图3:**
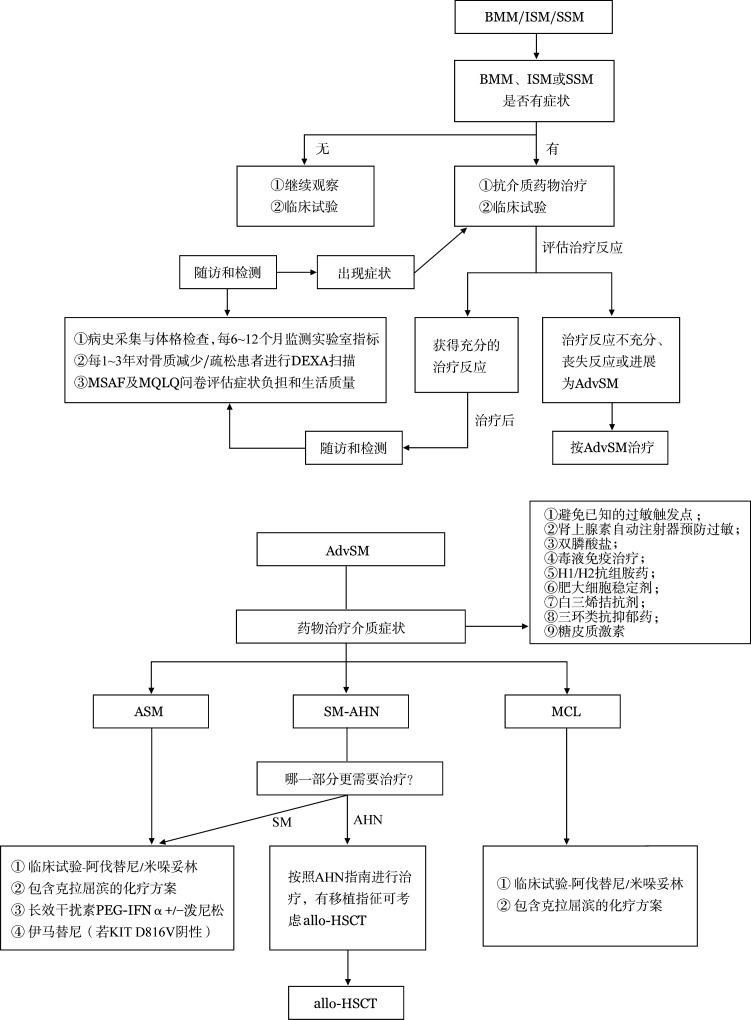
系统性肥大细胞增多症（SM）各亚型治疗流程 注 BMM：骨髓肥大细胞增多症；ISM：惰性系统性肥大细胞增多症；SSM：冒烟型系统性肥大细胞增多症；AdvSM：进展型系统性肥大细胞增多症；ASM：侵袭性系统性肥大细胞增多症；SM-AHN：系统性肥大细胞增多症合并相关血液肿瘤；MCL：肥大细胞白血病；SM：系统性肥大细胞增多症；AHN：相关血液肿瘤；PEG-IFNα：聚乙二醇干扰素α；allo-HSCT：异基因造血干细胞移植

（1）无症状惰性BMM/ISM/SSM：可进入临床试验或观察随访直到出现症状；

（2）有症状BMM/惰性ISM/SSM：可进入临床试验或抗介质治疗肥大细胞活化症状，评估治疗反应不充分、反应丧失或进展为AdvSM者，可参照AdvSM的治疗；

（3）ASM：首选临床试验或阿伐替尼/米哚妥林靶向治疗或allo-HSCT，其他可推荐包含克拉屈滨的方案、长效干扰素PEG-IFNα联合或不联合泼尼松，若KIT D816V阴性可选用伊马替尼；

（4）SM-AHN：以SM和AHN哪部分更需要治疗为原则，若SM更需要治疗，参照ASM各亚型治疗策略；若AHN更需要治疗，参照AHN指南治疗（包括考虑allo-HSCT及同时治疗SM）；若两者都不需要治疗，监测到新的C-发现后进行抗介质治疗。

（5）MCL：首选临床试验或阿伐替尼/米哚妥林靶向治疗或allo-HSCT，其他可推荐包含克拉屈滨的方案。

六、疗效评估

具体疗效评估标准见表7和表8。

**表7:** IWG-MRT-ECNM共识中ASM、MCL、伴髓系肿瘤SM的疗效标准

疗效标准	诱导缓解治疗
完全缓解（CR）：满足全部4个标准且持续时间≥12周	①在骨髓或其他皮肤外器官的活检中没有致密性聚集的肿瘤性肥大细胞；
	②血清类胰蛋白酶水平<20 ng/ml（仅治疗前>40 ng/ml时适用）；
	③外周血象缓解（ANC≥1×10^9^/L且外周血细胞形态正常，HGB≥110 g/L，PLT≥100×10^9^/L）；
	④可触及的肝脾肿大以及所有经活检证实或疑似的SM相关器官损害完全消失（CI症状）
部分缓解（PR）：满足全部3条标准且持续时间≥12周，未CR和疾病进展（PD）	①骨髓和（或）SM相关器官损害的其他皮肤外器官的活检中，肿瘤性肥大细胞减少≥50%；
	②血清类胰蛋白酶水平下降≥50%（仅治疗前>40 ng/ml时适用）；
	③有1个或多个经活检证实的或疑似SM相关器官损害得到改善
临床改善（CI）：持续时间≥12周，未CR、PR和PD	要求满足≥1个非血液学或血液学临床改善疗效标准（见表8）
疾病稳定（SD）	不符合CR、PR、CI或PD标准
PD：满足标准①或②中至少1项，且持续≥8周	①对于基线非血液学器官损害2级的患者：加重1级，且实验室异常指标至少增加100%；
	②对于基线白蛋白≥2级的患者：加重1级，且血清白蛋白水平降低≥5 g/L；
	③对于基线非血液学器官损害≥3级的患者：实验室异常指标至少增加100%；
	④对于基线非输血依赖性贫血或血小板减少≥2级的患者：在8周内新增输血需要≥4个单位红细胞或40个单位血小板；
	⑤对于基线输血依赖性贫血或血小板减少患者：与治疗前12周内相比，8周时间内平均输血频次增加≥100%；
	⑥对于基线中性粒细胞减少≥3级的患者：中性粒细胞计数减少>50%且中性粒细胞绝对计数减少≥0.25×10^9^/L且达到4级；
	⑦对于基线脾脏大小无法触及或≤5 cm者，可触及的症状性脾肿大至少增加10 cm；如果基线脾脏大小>5 cm，可触及的症状性脾肿大增加>50%且至少增加≥10 cm
缺乏反应（LOR）	未记录到超过8周的CR、PR或CI；
	降级（CR至PR或PR至CI）不能被视作LOR，除非降级后未达到CI超过8周；
	LOR的基线值是治疗前的测量值，不是治疗反应中的最低值

注 ASM：侵袭性系统性肥大细胞增多症；MCL：肥大细胞白血病；SM：系统性肥大细胞增多症

**表8:** IWG-MRT-ECNM共识中临床改善（CI）和器官损伤的评估标准

类型	器官损害	符合CI缓解评估条件的器官损害（CI症状）	CI的反应标准
非血液学反应	胸腹水	①有症状的胸腹水需要医疗干预，如使用利尿剂（2级）	①有症状的胸腹水完全消失，且不再需要利尿剂治疗≥12周
		②进入研究前12周内有≥2个治疗性胸腹腔穿刺，两次穿刺间隔至少28 d（3级），且其中1次在药物治疗前6周内完成。	②不需要胸腹腔穿刺术治疗≥12周
		注：满足①或②	注：满足①或②
	肝功能异常	①存在腹水时，DBIL、AST、ALT或GGT异常≥2级；	≥1项肝功能指标恢复至正常范围持续≥12周
		②临床相关门静脉高压症；	
		③活检证实肝脏肥大细胞浸润；	
		④不明原因的肝功能异常。	
		注：满足①和（或）②和（或）③或④	
	低蛋白血症	低白蛋白血症≥2级（即<30 g/L）	白蛋白水平恢复至正常范围≥12周
	有症状的脾肿大	症状性脾肿大：指脾脏触诊在左肋缘以下>5 cm，且患者主诉不适和（或）过早饱腹感的症状	触诊脾肿大缩小≥50%（也可行三维CT/磁共振成像评估检查），且不再主诉不适和（或）过早饱腹感≥12周
血液学反应	ANC	基线ANC≥3级（<1×10^9^/L）	ANC升高≥100%且ANC≥0.5×10^9^/L持续≥12周
	贫血（非输血依赖）	贫血≥3级（HGB<100 g/L）	HGB水平升高≥20 g/L持续≥12周
	贫血（输血依赖）	①在治疗开始前12周内至少输注浓缩红细胞6个单位，最近一次输注发生在前4周内；	脱离输血依赖持续≥12周，且在12周评价结束时HGB至少维持在85 g/L
		②在HGB≤85 g/L且不伴出血、溶血或其他治疗的情况下，将输注红细胞视为基线标准的一部分	
	血小板减少（非输血依赖）	血小板减少≥2级（<75×10^9^/L）	血小板计数升高≥100%，且PLT增加≥50×10^9^/L，且无需要血小板输注持续≥12周
	血小板减少（输血依赖）	①在治疗开始前12周内至少输注60个单位单采血小板；	脱离血小板输注持续≥12周，且维持PLT≥20×10^9^/L
		②在治疗前4周内至少输注20个单位单采血小板；	
		③仅在PLT<20×10^9^/L时输注血小板	
		注：需同时满足①②③	

注 疗效标准使用美国国立卫生研究院（NIH）的常见毒性标准（CTC）4.03版判定；IWG-MRT-ECNM：骨髓增殖性肿瘤研究与治疗国际工作组（IWG-MRT）和欧洲肥大细胞增多症研究网络（ECNM）达成共识认定的进展型系统性肥大细胞增多症标准[14]；DBIL：直接胆红素；ANC：中性粒细胞绝对计数
